# The Hospital Nursing Supplement

**Published:** 1892-07-09

**Authors:** 


### "The Hospital" Nursing Mirror.

Being the Extra Nursing Supplement of "The Hospital" Newspaper.

Contributions for this Supplement should be addressed to the Editor, The Hospital, 140, Strand, London, W.C., and should have the word "Nursing" plainly written in left-hand top corner of the envelope.

### En Passant.

HOLIDAYS.—A correspondent, "Jersey," will give nurses any information about Jersey, and recommends rooms at Mrs. Bienvenn's, 1, Watkin Villas, First Tower, where boating, bathing, walks, drives, and excursions may be had. The train leaves Waterloo at a quarter to ten p.m. week days, and a ticket for two months costs £1 18s. We will answer any questions we can.

CHELTENHAM GENERAL HOSPITAL.—It has been decided that the private nursing staff at this hospital shall be increased from six to twenty, and a new home is to be built for the nurses; the only objection to the idea was that it would injure an already existing private institution in the town, but it is thought that there is plenty of room for both.

THE HOLBORN GUARDIANS.—It has been proposed that this Board should employ probationary nurses who would sign for a period of three years, and thus the Board would ensure themselves from continually changing nurses; but the proposal, we regret to say, was lost because the Medical Superintendent did not want the arrangement. As it is the assistant nurses are not trained, and when they think they know a little they depart, but the Board failed to see any use in turning the Highgate Infirmary "into a school for young nurses," so what more can be done? We can only suggest a visit to Marylebone Infirmary, Notting Hill.

SHORT ITEMS.—The Staffordshire Institute for Nurses have had an excellent year of work, and a sum of money has been placed at Miss Shirley's disposal for division among the nurses according to a scale she has prepared. —Steyning is still afflicted with disputes; the Matron and Head Nurse are endeavouring to get on together.—The Ballymena Nurses have done well, Miss O'Neill is now the Lady Superintendent.—Kinsale Infirmary is to be nursed by nuns in future, and the Board of Guardians have voted £669 to erect a Nurses' Home; it is pleasant to find the Local Government Board has not made its suggestions altogether in vain.

PRIZE-GIVING AT SCHOOL OF MEDICINE FOR WOMEN.—The distribution of prizes at the School of Medicine for Women, by the Duchess of Bedford, took place on June 28th. There was a large gathering on the occasion, and many of the guests congregated in the garden, hearing the speeches through the open windows of the hall, which only afforded accommodation for a portion of the company. The Dean, Mrs. Garrett-Anderson, spoke of the satisfactory financial position of the College, and of her confidence that the day was not far distant when a handsome and suitable building would replace the present makeshift arrangement. The Duchess of Bedford made a very interesting speech, and showed the keenness of her interest in woman's education and welfare. Mrs. Peachey-Phipson entertained her hearers with a charmingly graphic picture of the prosperous school of to-day, with its hundred students, in contrast with the time—nearly twenty years ago—when she, Mrs. Thorne, and five other ladies passed their examinations in Edinburgh, in the face not only of verbal abuse, but of worse insults still at the hands of the "nobler" sex, though even then a few gentlemen stood forth as their champions, and defended and encouraged their right to choose their own career.

THE Q.V.J.I.N. IN SCOTLAND.—Nowhere is the work of this national undertaking making better progress than it is in Scotland; we are constantly hearing of new branches; the family of the late Mr. and Mrs. Hugh Rose, of Edinburgh, have placed capital in the hands of the Scottish Council to endow a District Nurse for the districts of Canonmills and Broughton, Edinburgh. Hamilton is to have a branch, and the Hon. Miss Hamilton, Dalzell, is appointed President. News come from Montrose that a grant to the local branch will, it is hoped, be given by the Trustees of the Cobb bequest; if it is forthcoming, this will enable the Committee to start another nurse at work, whose services will be very welcome. It is very encouraging to find so much appreciation of the work, and to hear of the continuous efforts made to add to its usefulness.

NURSES FOR INDIA.—We hope our readers will notice the interesting letter sent to us this week by Mrs. E. L. Robertson. Many of our readers may not have seen the article which appeared in August last year in the *National Review* written by this lady, which describes the social condition in the large Indian towns of those who are nothing less than our poor fellow-countrymen, the "submerged tenth," in fact, of Anglo-Indian society, the descendants of Englishmen who, in the struggle for existence, have sunk to a state we over here can scarcely realise. Mrs. Robertson pointed out that while the poorer natives are being cared for morally, medically, and socially, there is a vast population of English and Eurasians practically uncared for. It is in securing adequate medical relief for this class and by supporting and enlarging the scheme of existing hospitals in India that Mrs. Robertson hopes nurses in the future will become available for all sorts and conditions of men in India.

INVALID CHILDREN'S AID ASSOCIATION.—The annual meeting of this society was held on June 30th, and was a very interesting gathering. Sir Charles Freemantle was in the chair, and Mr. Timothy Holmes and Dr. Broadbent gave great praise to the system of relief carried out by the Association. As a means of communication of sympathy between rich and poor, this Association can hardly be bettered, and it seems to us the realisation of true charity to find those who can bring alleviation of suffering to sick, poor, and helpless little children, working so quietly and unobtrusively, by personally ministering to their wants, brightening tedious days by kindly visits, and becoming in fact the "friend in need" so many of us have felt the want of at some time or another.

STALYBRIDGE SICK NURSING AND MOTHERS' HELP SOCIETY.—We are glad to receive the last report of this Society, and to see that its eleventh year of existence finds its useful work increasing. In November, Nurses Twamley and Farrand will have completed eight years' work for the Society. The former is the district nurse, and she paid 4,922 visits during the year; while Nurse Farrand, the maternity nurse, nursed 71 cases. In Miss Mansell's opinion, these nurses have attained to an unusual standard of excellence in their profession. The Committee have received numbers of letters in grateful acknowledgment of the nurses' services and kindness. A third nurse is now at work, and only financial help is needed to further extend the work. The system of lending out appliances for a small weekly fee, such as bath chairs, hot-water bottles, water beds, &c., seems an admirable one; for instance, the charge for a bronchitis kettle is 4d. a week, and bed linen is also lent out at the same reasonable rate. The year's expenses were only £158.

### Ventilation, Disinfection, and Diet.

By P. Caldwell Smith, M.D.

### XIII.—DIET AND DIETARIES.

Construction of a Diet Table—Relation between the Nitrogen and Carbon—Energy Derived from Foods—Vegetarianism—Composition of some Articles of Food—Meat—Beef—Veal—Mutton.

For the construction of a diet table it is necessary to refer to tables in which the different amounts of these principles are given. To use these tables only involves the knowledge of simple proportion; the quantity of bread and meal is given; and then the proportions can be brought out.

Suppose, for example, we want to know if 2lb. of bread and ½lb. of cheese are sufficient for a man at moderate work, on looking up a table containing the amount of proximate principle in these we find that 100oz. of bread contain 8oz. nitrogenous, 1½oz. fat, and 50oz. carbohydrate; ∴ 32 oz. or 2lb. contain 2·5oz. nitrogenous, ·5oz. fat, and 16oz. carbohydrate; 100oz. of cheese contain 33oz. nitrogenous, 24oz. fat, and 0oz. carbohydrate; ∴ 8oz. or ½lb. contain 2·6oz. nitrogenous, 1·9oz. fat, and 0oz. carbohydrate. Adding these together we find that the total amount is 5oz. nitrogenous, 2·4oz. fat, and 16oz. of carbohydrate.

Now, on looking at the table previously given, you will see that the amount there stated as average for man on moderate work is 4.5 oz. of nitrogenous, 3oz. fat, and 15oz. carbohydrates, or that the bread and cheese would be quite sufficient, as far as the nitrogenous and the carbohydrates were concerned, but would be deficient in fat.

It is not enough, however, in calculating a diet theoretically, to put together a number of nutritive substances and find by a table their value. If one had to live on calculated diets, it would become decidedly monotonous, so there must always be variety. That is one reason why fruits, vegetables, &c. are so pleasant; they are not only antiscorbutic, or preventive of scurvy, by they also, to some extent, help to variegate a diet, which would without them be monotonous.

A very important thing in a theoretical diet is the relation between the amount of N. and the C, the best relation being 1 of N to 15 of C, or the amount stated in grains, 300 grains of N to 4,500 of C. A large number of experiments have been made with the different kinds of foods to find out the amount of energy which should be derived from these, and tables have been formed showing these amounts in what is called foot-tons. It is to be kept in mind that these only express the theoretical energy, for although we can say, in the consumption of coal or oil, how much work should be produced, yet in the case of the human body the combustion which takes place is by no means analogous. For example, in the case of nitrogenous food a large amount of it is never oxidised at all, but passes out of the kidneys in the form of urea, while sugar and starches are almost completely oxidised. This will be seen by further reference to the table in which the amount of food passing away unutilised, varies from 4 per cent. in the case of rice to 50 per cent. in the case of gelatin.

Take again the case of fats. Outside the body nothing is more combustible than the various forms of fats. Put a piece of beef fat in the fire and it burns briskly. In the human body, however, fats are very slowly oxidised. It would be a great mistake to form a diet according to this potential energy. As it has been found that substances possessing little dietic value have as much potential energy as those possessing a large amount.

It may be interesting at this point to say a word regarding one of those fads which some people swear by—I mean that of vegetarianism. The more advanced of these not only eschew all forms of animal food as beef, mutton, veal, fowl, game, fish, &c., but also milk, butter, cheese, eggs, &c., while the great majority of them still continue to consume these latter. One great fault about a vegetable dietary is that it is apt to contain too little nitrogen. The great advantages of animal food are that they supply, in a much more convenient form, what is required to make the colouring matter of the blood. The fats derived from the animal kingdom, as for example butter, are very much more digestible than those derived from the vegetable kingdom. Take, for an example, the low class Indian, who hardly ever takes any other thing than rice; the amount of nitrogenous material in this is extremely small, and to take enough to make up the physiological amount he would require to take an enormous quantity, and in this case the amount of carbohydrates would be more than he could digest. To make up, however, for the want of this in rice he takes some lentils, which contain a large proportion of albuminous substance, and so the equality is kept up. It is not always safe to generalise from individual instances, and although one man may be perfectly healthy and vigorous on a so-called vegetarian diet, yet that is not to say that we should all abstain from eating meat. I am inclined to think that wherever we have civilisation we have extremes, in eating as well as in drinking. On the one hand you have the rabid teetotaler and the vegetarian, while on the other you have the drunkard and the glutton. There is also no doubt that most people eat more nitrogenous material that is really required, and this is the cause of many cases of so-called biliousness, of gout, and diseases of liver and kidneys. The happy mean is best, and if we take care not to exceed, or not to exceed very much, the physiological limit, we will have a great deal more bodily and mental vigour than most so-called vegetarians.

Let us now look for a little at the composition of some of the more common articles of food.

1. Meat: This includes beef, veal, mutton, lamb, pork, game, fish, fowl, &c. Beef has always been regarded as by far the most nutritious form, and it contains about 75 per cent. of water, 20 per cent. of albuminous matters, including all the albumin in the flesh itself, and the gelatin in the cartilage or gristle. This proportion of flesh-forming material is greater than is found in mutton or bacon. As it has a closer and firmer fibre than most other kinds of meat, it is, bulk for bulk, more nutritious. Other forms of meat do not to most people possess such a rich flavour as beef, and for these reasons the consumption of it is much in excess of all other kinds. Beef varies in quality according to the age of the animal from which it is taken, as well as its mode of feeding. The proportion of fat to the whole animal is about 33 per cent., or one-third. This is found chiefly on the inside of the loin, from which it is sold as suet, also on the thin ribs, while in fat animals it is all over the body. In cooking beef there is less loss of weight than in cooking mutton. Consequently it is much more economical.

Veal is not a meat that should be much eaten; at least, when the animal from which it is taken is under two months old. In this country a large amount of veal is consumed, from the calves killed immediately after birth. On the Continent, on the other hand, they are not killed till they are six or nine months old.

Veal is difficult to digest, owing to the length of its fibres and their liability to escape mastication. It is said to be more easy of mastication when roasted or broiled. Calves-foot jelly is, as you know, used very much for invalids, and the reason why it is used is that the bones of a young calf contain a large proportion of gelatin and little earthy matter.

Mutton does not contain so much nitrogenous matter as beef, while it contains a larger proportion of fat. It is, therefore, bulk for bulk, not so suitable for the production of energy, but is specially useful for invalids.

### Select Committee on Metropolitan Hospitals.

(From a Nurse's Point of View.)

At last the report of the Committee of the House of Lords lies before us, and, like most other things in this world, it fails to give universal satisfaction. The nursing section has evidently the prior claim for discussion in "The Mirror," and it is with that we propose just now to deal. The suggestion that each nurse should be entitled to two whole days off duty in a month will commend itself to many a probationer, although our own bias is towards twenty-four hours' leave of absence once in the month, as a more satisfactory arrangement. Also we must put forward the fact that there need be a large increase of nurses in most of our general hospitals to meet this demand, for the present custom of letting the others share between them the extra duties of the absentee ought not to hold good when days off duty are doubled. The much discussed London Hospital has always given to staff nurses and probationers two hours daily off duty, and classes for instruction and lectures are included in the day's work. At most other hospitals this has not been the practice, although we regret to say public attention has not investigated the reason for such doubtful proceedings as "classes," "choir practices," and "lectures" being abstracted from a nurse's nominal "recreation." It is a pity that some experienced and practical-minded nurse was not consulted as to fair working hours in the course of this lengthy, though hardly exhaustive enquiry. She would tell us that eight hours' work is not sufficient to satisfy any normally healthy woman; she would also remind us that many who come from the provinces are without friends in London on whom they can bestow their society in their intervals of leisure. Free sights are exhausted in time, and to pay for admission to places of amusement can only be an occasional indulgence. Parks and gardens are summer joys only, and sitting out of doors is an uncertain pleasure during nine or ten months in English weather. These reflections tend to bring us to the nurse's sitting-room as a haven of refuge, but we also remember that only a small proportion of women devoted to nursing are really students, and the recreation-room is apt to become an undesirably gossippy headquarters. We maintain that much of the work done for the sick becomes mechanical, and, therefore, ceases to cause any such severe mental strain as outsiders suppose it to do. Even the care, watchfulness, and sympathy characteristic of the true nurse grow to be so entirely natural that they are exercised without conscious effort. Albeit some days may be full of wearing excitement and fatigue, there are many when no such demand is made on a nurse's powers, and she is not a bit the worse for spending ten hours in the company of those to whom she can minister instead of sitting in idleness and discussing many things which, if not harmful, are distinctly unprofitable to her moral being. We do not think a bare third of her time is a sufficient proportion, nor one that will content any earnest worker amongst the sick. There is more practical application in the proposal that a Matron or her representative should dine with her nurses; in some institutions this arrangement always prevails, but decently cooked and nicely served meals for nurses would be no longer the exception instead of the rule, if a *fair* report of the food were asked for and encouraged! Generally the report has to be made up by an assistant or underling of some sort, and when he or she makes unfavourable remarks on any provisions, the person to whom it is delivered shows plainly that such statements are unwelcome, and not unfrequently such a close catechising results, that the would-be honest assistant is apt to take refuge in silence on the next similar occasion, feeling either that it is "no use to speak," or else that he will harm himself and endanger his position by being dubbed a malcontent. If other things in hospital administration had made as good progress during the last few years as the Pension Fund has done, there would be little need for further reforms. On that point we venture to speak with much comfort. As regards private nurses we think the "Lords" do well in advising a separate staff of fully trained nurses, but we think that higher annual pay and longer holidays give better results than the paying of commission, however arranged. Matrons have at all times most difficult work to do, in fact *how* difficult it is for them to hold the reins of government justly no one outside a hospital can fairly judge. Their difficulties will be trebled if they are to have no longer power to deal with unsatisfactory probationers; we think that questions connected with sisters and staff nurses may well come before a committee, but is it fitting that this body should be composed only of men if it is to deal with such absolutely womanly matters? If the committees already established, and doing, many of them, work which the Lords have specially appreciated and commended, show themselves unwilling to recognise the necessity for admitting women to their boards, they might yet be not averse to the appointment of a Ladies' Sub-Committee (or executive) to carry on such business as is specially connected with nursing matters. A Ladies' Committee in days gone by was of little use, save in name, but these things have changed, with many others, and a great deal of sound business is got through by quiet boards of which the world hears nothing. We are very much delighted by the attention of the public being called to the absolute necessity for trained Matrons in Poor Law infirmaries, and for the supply of proper nurses in sufficient numbers, and we may add our own hope that it will not be very long before each general and special hospital in the kingdom is under the supervision of a trained Matron. Surely, it is a serious blot on hospital legislation that the post of Superintendent of Nurses should be filled by unqualified persons, especially in nurse-training institutions.

We note, with pleasure the strong advocacy for convalescent homes in the Lords' Report, and we trust that we may some day see each hospital provided with its own seaside or country establishment, to which all patients needing and desiring the benefit of a change of air shall be sent. These places are of wonderful benefit in perfecting a cure and even in hastening convalescence, but they need more careful supervision than is always accorded to them, as also more provision for the comfort of patients transferred to them after weeks or months of careful nursing in a ward, and who are still practically incapable of doing as much as is too often expected from them. Here, again, is a case where a trained Superintendent and nurse become absolutely necessary for the well-being of the invalids, and it is folly to imagine that patients nominally convalescent are in no need of skilled care. All of us could cite cases of hospital patients who have had relapses at convalescent homes, and although some instances may be unavoidable, others must certainly go to swell the lists (already large) of "preventable diseases." We are favourably impressed by the Lords' strong recommendation that a Ladies' Committee should be appointed to help in the much-needed reforms at the Royal Hospital for Incurables at Putney. This would certainly prove a good step in the right direction, if the members of it are wisely chosen, for it is very necessary that women should be thoroughly conversant with the practical details of hospital work and management before they attempt any legislation on this important subject.

### How to Procure Nurses for India.

*To the Editor of* The Hospital.

Sir,—The following remarks are suggested by two letters which appeared last April in *The Times*, on the subject of "Nurses for the English in India."

Both writers draw attention to the sufferings of the Anglo-Indian community for want of proper nursing, and indicate the same remedy, that of supplying the need by means of professional nurses trained in England. "Edith M. Cuthell" opens the subject by referring to the congested state of the labour market for women in England, and pointing to "the important opening in India," where she considers "there is a prospect to any certificated and efficient nurse of making a very good livelihood." She adds, "the pay is excellent, the demand is large."

Dr. J. Butler Hamilton, in his review of her letter, takes a less sanguine view of the financial prospect. He says, "The demand is great, but the pay, I regret to say, is *not* excellent." He compares the large fee, which, in their own interests, professional nurses would be compelled to ask, with the modest salaries of the younger officials, even of the best paid classes, and concludes that the scheme will not "pay" unless it is established on a semi-charitable foundation by the aid of "a national subscription."

Both the writers whom I have quoted make special reference to the scarcity of nurses in the North-Western Provinces, and as that part of India is very familiar to me, and I feel deep interest in the subject, I venture to offer a few criticisms on their views in the hope of throwing fresh light on the subject.

The dearth of skilled nursing is a fact which cannot be disputed, and it is scarcely necessary to add that much preventable suffering and loss of life is the inevitable penalty, especially amongst the young, whose inexperience makes them an easy prey to the dangers of the climate, and who are willing to run all risks for the sake of amusement.

The real difficulty is to suggest a remedy for all this suffering; and perhaps some practical information may be gained by considering why, when such a bitterly felt and universally acknowledged need exists, Anglo-Indians themselves have done so little to relieve it. In the first place, we must bear in mind that trained nurses in England are the natural outcome of an admirable system of hospital management which exercises the double functions of relieving physical suffering and of providing a training ground for medical science and nursing. Looking at the N.W. Provinces and other parts of Upper India in the light of this experience we discover that although suitable material abounds, the training grounds do not exist. Need we therefore go further to discover why Anglo-Indians, including those willing and able to pay, have never been able to provide themselves with good nurses?

For many years past there have been hospitals in every large town and village, maintained mainly by Government on a meagre scale of comfort for natives of the poorest class, but the question of nursing has never been taken into account, the nearest approach to it being the admission of relatives to nurse those patients too ill to look after themselves. In few or any of them will be found the most elementary conveniences for the reception of patients of European habits, who, in fact, do not resort to them.

So lately as two years ago there was only one European hospital in this province, and the staff did not include a single permanent nurse, one being engaged (if she could be found) when it was considered necessary.

Happily, this condition promises to improve. Owing mainly to Lady Dufferin's efforts on behalf of native women, much light has been thrown on the shortcomings and insufficiency of hospital relief for the poor; and female hospitals, at least, are already greatly improved, resulting in a large increase of patients and a corresponding demand for the services of nurses. For instance, in the large station of Allahabad, quoted by Mrs. Cuthell, a well-organised hospital for all races and conditions of women was opened last year, and classes for the instruction of nurses promise to supply better trained material than "the few sergeants' wives, mostly Eurasian," on whom, as Mrs. Cuthell truly says, they have hitherto depended for nursing. In Naini-Tal, about the same time, a hospital was opened for both sexes and all races, and if this noble work, which languishes for lack of funds, receives the sympathy and support it merits, it cannot fail to advance until the supply of both hospitals and nurses, in some measure, overtakes the demand. It must not be forgotten that amongst the many millions of human beings in North-Western India a mere handful realise the benefits of hospitals and trained nurses. Although the influence of the Dufferin fund may have helped to open the eyes of the natives to the value of these blessings, our system of medicine has really made very slow progress amongst them, and all but a fraction still adhere faithfully to the time-honoured prescriptions of their own Hakims. In the present state of their knowledge they cannot be expected to give cordial or spontaneous support to charitable hospitals. Europeans in India, as most of us know, are chiefly officials or professional men. Never rich at any time, their incomes are now grievously reduced by the depreciation of silver, and they are, as a rule, very poor indeed.

Would it not, therefore, be infinitely more practical to support, by every possible means, this movement for improving and extending hospital relief for the poor, and thus to create in the country a training ground for nurses, rather than to import them from England at such a cost that they would be available to only a few at the top of the social tree, leaving untouched the mass of suffering below?

Although the two writers whose letters I have quoted, entitle them "Nurses for the English in India," they have evidently not taken into account the needs of that large portion of the Anglo-Indian community, who can pay very small fees, still less of the miserable dregs of the population, the alleviation of whose physical sufferings is entirely dependent on the humanity and charity of their more fortunate fellow countrymen.

It is greatly to be feared that the professional nurse, trained in England, cannot be produced in India at popular prices, and that those of this class who depend on fees for a living, will find their sphere of usefulness greatly restricted. As Dr. Hamilton has pointed out, the highest remuneration they can hope to receive will be but a poor return for the initial expenses of outfit and journey, cost of living in India (which should include a substantial margin for periods of enforced idleness), to say nothing of the wear and tear of health and strength consequent on arduous work in a trying climate. All this should be taken into account by nurses who look to India as a possible career. The extension of hospital relief will improve their prospects of employment, and a few nurses of a superior class will find their true vocations as Matrons of such establishments, in which position they could utilise their skill for the instruction of local talent, thus greatly increasing their own value, and adjusting the financial balance with regard to their cost. In conclusion, it cannot be too clearly realised, with regard to this province at least, that the dearth of nurses in the upper ranks of society is the natural result of long-neglected obligations towards the physical sufferings of the class who, in all civilised countries, are provided with *gratuitous* nursing in the wards of a charitable hospital. Any scheme, therefore, which proposes to supply nurses to the paying portion of the community, irrespective of the crying need which lies at the root of all the suffering, will prove a very superficial remedy, too costly in proportion to its utility.

If English benevolence will come to the aid of charitable hospitals in North-Western India, in order to provide suitable accommodation within their walls for the poor waifs and strays of our own race who, with their descendants, are stranded on those distant shores, the latter will prove valuable material for the clinical teaching of nurses so urgently needed by those of our birth and kin who are serving in India.

We shall then fulfil the obligations of humanity, and find a practical solution of the problem how best to supply "Nurses for the English in India." E. L. Robertson.

### Asylum Notes.

CANDIDATES WHO PASSED THE EXAMINATION OF THE MEDICO—PSYCHOLOGICAL ASSOCIATION FOR THE CERTIFICATE OF PROFICIENCY IN NURSING, MAY, 1892.

Haywards Heath Asylum—*Males:* John Backhouse, Albert George Wake, George Catchlove, Timothy Callaghan, James M. Le Patourel, Jasper H. Smith, and Frederick Cook. *Females:* Emma Ware, Kate White, Mary Alice Warner, Caroline M. Walton, and Alice Ann Derham.

Birmingham Asylum.—*Males:* Henry Ambrose Hill, Peter Warburton, and Harry E. Drew.

Wadsley Asylum, Sheffield.—*Males:* Robert Barwell, Harry B. Ellis, Herbert Brooke, Thomas George Harrison, William Severns, James Dolan, Abraham E. L'Amie, Joseph Ashby Dixon, Arthur W. Redfern, Nelson Webster, John Moore, Albert W. Jones, and Shaw John Davies. *Females:* Flora F. Drabble, Ellen Healey, Mary Madeley, Christiana Emmerson, Elizabeth Cullabine, Fanny Wheatley, Annie Marks, Lizzie Cousins, Elizabeth Frazer Scott, and Eliza Robinson.

West Riding Asylum, Wakefield.—*Males:* William Reed, Eyra Frost, Richard Steele Robinson, James Arthur Hadfield, George Hobbs, Richard Howden, John Whipp, James Wright, and Henry Wright.

Rubery Hill Asylum.—*Males:* Joseph Storer Noon, and William Phillips. *Females:* Kate Cocks, Nellie Shields, Emily Worrall, Edith Cutler, Martha Sayers, Emily Taylor, Sarah Bishop, and Emily Arundel Withers.

Northumberland County Asylum.—*Males:* George Baker, Robert James D. Brown, Thomas Flanagan, and William Philipps. *Females:* Frances Jane Richardson, Susannah Thompson Palmer, Elizabeth Crake, and Melville Annie Armstrong.

Holloway Sanatorium.—*Males:* A. Pratt, Henry James Lee, John McLaren, and George Wright. *Females:* Ann Raw, Annie Cornaby, Annie Oliver, Jane Chapman, and Frances Matheson.

Roxburgh District Asylum, Melrose.—*Males:* David Anderson and Gilbert Millar. *Females:* Mary M. Reid.

Gartnavel Asylum, Glasgow.—*Males:* John Nicoll, John Wallace, James Thornton, James Durward, and Duncan Urquhart. *Females:* Helen Begg, Margaret Innes, Christina Robertson, Helen Sutherland, Jane Barr Gray, Margaret Stewart, Isabella Gillies, Harriett McIntyre, and Margaret Hendrie.

Dundee Royal Lunatic Asylum.—*Males:* Thomas Boyd, James Inches, and Charles Chalmers. *Females:* Jane S. Alison, Joan M. Finlayson, Jane McGarrock, Sarah McGarrock, and Jessie Shand.

At a special meeting of the Medico-Psychological Association, held at Bethlem Hospital on Thursday, June 23rd, 1892, it was resolved:—

1st. "That there be a Committee of Education temporarily appointed, consisting of Members of the Association who are teachers of Psychological Medicine in the Universities of Medical Schools in England."

2nd. "That the Committee be instructed to take steps to be represented before the Commission of the New (Gresham) University."

3rd. "That this meeting recommends to the annual meeting that a Board of Education be appointed to consider all questions affecting medico-psychological teaching, the Board to consist of all members of the Association who are lecturers and teachers of psychological medicine in the universities or medical schools of the United Kingdom."

### Everybody's Opinion.

[*Correspondence on all subjects is invited, but we cannot in any way be responsible for the opinions expressed by our correspondents. No communications can be entertained if the name and address of the correspondent is not given, or unless one side of the paper only be written on.*]

### NURSES' HOLIDAYS AND KING'S COLLEGE HOSPITAL.

"Sister" writes: I see in the report of the Lords' Commission, published in your paper of last week, that Guy's is mentioned as giving the longest holidays to their Sisters and nurses. Will you allow me to state in your columns that for more than three years the Sisters at King's have had six weeks' holiday, the staff nurses a month, and the probationers three weeks, besides extra leave in case of sickness either among ourselves or near relations? Our off-duty hours are longer than those of any other hospital. It seems only just that when so much has been done by our Matron for her nursing staff that the public should be told of it.

[We very gladly publish this letter. It was a heavy task for the Lords' Committee to digest all the evidence, but "Sister" will see they found that nurses' holidays vary "from a fortnight to a month," which is correct. Sisters ought to have more—six weeks at least. No one can visit King's College Hospital without being impressed with the ability and success of Miss Monk's administration and system.—Ed. *T.H.*]

### THE HAMILTON ASSOCIATION FOR MALE NURSES.

"The Medical Superintendent" writes: I should like to be allowed to correct an error in your appreciative notice of our work. On referring to our rules you will see that a guinea a day is not our ordinary charge, but the special fee for removing an insane patient. The two rules to which you object are almost verbally identical with the two in the prospectus of the nursing institutions in connection with two of our largest hospitals. The Committee have never withdrawn a nurse from a patient, but it is obvious they must retain the right to do so.

### JERSEY.

"Norfolk Matron" writes: I shall be glad to answer all particulars as to Jersey and Guernsey. I know both places, and I think I know the cheapest route. I should like to join should a party be made up and the date suited.

### Queen Victoria's Jubilee Institute for Nurses.

Her Majesty has been pleased to approve of the following additional names being entered on the roll of Queen's Nurses for nursing the sick poor in their own homes: Superintendents—Florence Saunders, Mary Elizabeth Sedcole, and Ellen Mary Lush. Nurses—Margaret Anderson Rose, Edith Scott, Jane Battersby, Janet Mildred Baskett, Alexandra Chadwick, Isabel B. Wilson, Edith Mary Swainston, Matilda Veale, Grace Penrose, Margaret Penman, Jeanie Jones, Elizabeth Hudson Courtenay, Amy Judd, Fanny Lewis, Cecil Eleanor Wrixon Becher, Eleanor Terry, Mary Sophia Bryan, Elizabeth Marris, Mary Adelaida Ross, Katrina Knight, Dorothy Albinia Lancaster, Kathleen Ryan, Margaret Davies, Florence Forrest, Elizabeth Cleeve Hosking, Annie Bevan, Lucy Sarah Codrington, Laura Sophia Davies, Mary Judson, Anatolie M'Cully, Jessie Maria Nelson, Selina Sellers, Mary Ann Stephen, Mary Teresa Swift, Susan Wilson, Annie Bessy White, Euphemia Kirk Philp, Mary Macmaster, Lilian Sarah Jameson, Emily Rebecca Terry, Mary Pringle, Elizabeth Jessie Wilson, Agnes Hanna Brock Hadden, Margaret Dawson Greig, Mary Carlile, Leah Garratt, Wilhelmina Low, Kate Porter, Alice Hinton, and Margaret Taylor.

### Wants and Workers.

*The Matron of the Victoria Infirmary, Northwich*, would be very grateful for a Thomas's splint, 32 inches long, for a poor child whose parents are too poor to procure one. Carriage will be paid willingly.

*Nurse Naomi, of the Institute for Nurses, Lincoln*, would be grateful for a spinal carriage which would enable a poor cripple to get out into the air, who is otherwise obliged to be always in bed.

*District Nurse, Bangor*, is grateful for "Good Tidings" from Southampton.

### Four Months in a Hospital Ward.

*(Concluded from page c.)*

*(Concluded from page c.)*

### The Chapel.

On the first Sunday, after I had been allowed to go down stairs I attended the hospital chapel for the first time. On one side of the chapel sat the matron, doctors, nurses, ward-maids and porters, and on the other the patients, the intervening space being filled with the wheeled chairs of those of us unable to walk. The service which was conducted by the hospital chaplain, consisted of a portion of the evening service, three hymns, and a short sermon, the whole occupying about an hour. On some evenings I used by permission of the charge nurses of other wards, to pay short visits to patients in their departments, and in this way extended my circle of hospital acquaintance most agreeably. One of the charge nurses was musical, and encouraged her convalescents in singing part songs, sacred and secular; this select coterie I was invited to join and many a pleasant evening hour was passed thus. At about this period one of the nurses showed me all over the hospital, excepting of course the "fever block," and everywhere I found the same scrupulous cleanliness and order as existed in our own ward, and the same cheerful resignation marked in the faces of the sufferers as already mentioned in connection with my own companions.

### "The Children."

One department made a vivid impression upon me, for although my eyes had now grown accustomed to hospital scenes, and I could look calmly upon even the agony of death, I felt almost hysterical on leaving that most heartrending of all scenes, the suffering of helpless, innocent, childhood. It was the children's ward; they, poor infant mortals, were nearly all asleep on the evening of my visit, and happily oblivious of their early troubles; but I could not put away the thought of those other yearning hearts to whom no real sleep would come until their darlings were safe back again under the shelter of the maternal wing. And, oh! how old some of their faces looked; here upon the countenance of a child of five years of age could be seen the anxious lines of an overworked woman of twenty-five. What has done it? It is a bad sight to see a strong man suffering, but, oh, it is a thousand times more grievous to watch the restless tossing of a little child in weary, weary pain, that you are utterly powerless to shield it from. From the charge nurse I learnt that they have the utmost difficulty with the mothers on "visitors' days," and I can well imagine that it is so. One most exasperating case I saw of a child about four years old, who had been deliberately frightened by some senseless fool of a boy; a well-formed, well-nourished, promising little fellow, as completely snuffed out as if he had been killed; indeed, it would have been better so, for nothing remains to that poor waif now but the horrible existence of an idiot. Look, ye mothers! and see that an elder boy is soundly thrashed whose sense of humour can find no better expression than the frightening of his little brothers and sisters.

### "I am Discharged."

It was now about the end of my fifteenth week, and at last the doctor signified his intention of giving me my "discharge" on the next board day. I was transported with joy and could hardly believe it was true; to the doctor I tried to convey my sense of gratitude to all connected with the hospital for the inexpressibly humane manner in which, from first to last, I had been treated, for never one harsh sentence was addressed to me during all those long months; but words were too feeble to convey one half of what I really felt. Board day came, and a little group of us were assembled in the chapel to await our turn before the Governors. I felt a little nervous on being ushered into the board-room, where I found myself in the presence of some half-dozen gentlemen seated at a table. The Chairman, in a kindly tone, asked me if I was better, to which I replied in the affirmative and thanked him, he then asked me if I had been kindly treated, and I said "exceedingly," at this he handed me a paper of "discharge," and telling me to give it to the subscriber from whom I had obtained my hospital notes, bade me "Good morning," and the ceremony was over.

### "Good-bye."

Nothing now remained but to bid adieu to my companions in the medical ward; not an easy duty, for it tinged with sorrow the joy I felt at my own deliverance, and when the dying acrobat, giving me his now almost nerveless hand, said with quivering lip, "Good bye, and good luck go with you," I came very near breaking down. A few words of heartfelt thanks to that most estimable of women, our "Charge Nurse," and I found myself passing out through those iron gates which four months ago I had entered in such fear and trembling as a "forlorn hope." There was joy at home that day you may be sure, and when you consider that I am but one of the many thousands of bread-winners this single institution has restored whole and sound to his responsibilities, and who, but for its timely assistance, must surely have died, you will agree with me that of all the charitable objects presented to our notice, there is no channel so pure and so worthy of our best efforts as that of a hospital, where the only qualifications necessary to be a recipient of its bounty are that you be ill and in need.

### An Appeal.

If there be any who, up till now, though able, have never given their support in this direction, to such I would say, go and visit these institutions; not only on grand days, when some illustrious personage is inaugurating a new wing, but quietly on some leisure afternoon, and see for yourself the grand work of charity that for years has so unassumingly been carried on almost at your very doors. Speak to the patients and hear what they will have to tell you of what is being done for them. See the glad smiles of the nurses as with just pride they point to this or that convalescent whom they have watched day and night for weeks, through the acute stages of some deadly illness, and who count that convalescence a rich reward for their labours. Do this, and I am much mistaken if the interest in your suffering fellows thus awakened will ever be allowed to wane, for here will be a revelation of what the Psalmist felt when he said "Blessed be the man that provideth for the sick and needy."

### Notes and Queries.

To Correspondents.—1. Questions or answers may be written on post-cards. 2. Advertisements in disguise are inadmissible. 3. In answering a query please quote the number. 4. A private answer can only be sent in urgent cases, and then a stamped addressed envelope must be enclosed. 5. Every communication must be accompanied by the writer's full name and address, not necessarily for publication. 6. Correspondents are requested to help their fellow nurses by answering such queries as they can.

### Answers.

*Bob.*—We cannot remember what watches they were you mention, but you could get a good "Waterbury" watch for that sum, and they wear and work splendidly.

*Nurse W.*—The Secretary, Ambulance Department, St. John's Gate, Clerkenwell, will supply you with the information.

*An Aged Nurse*—Why have you never written again? You give neither name nor address, so we are powerless to help you.

*K.P.*—Apply to Matrons of good provincial hospitals. We do not think you can enter any London Hospital in the way you propose. If you are willing to go through a regular training for three years, apply for probationers' rules to King's College Hospital, the London Hospital, or Royal Free Hospital, Gray's Inn Road, or St Pancras Infirmary at Highgate.

*Patience.*—We will make enquiries for you, but we do not think such a society exists; could you not get on better in England as an obstetrical nurse? You are in the land of good nursing where you are, and could get information in the City. Will you perhaps write to "Nursing" if we can help you; look out next number for another reply.

*Beef*—As you do not mention what particular extracts you have bought we really cannot answer, but instructions as to doses will be found with all bottles. It must be obvious to anybody that "too much" can be taken of anything.

